# Revealing teaching quality through lesson semantics: A GPT‐assisted analysis of transcripts

**DOI:** 10.1111/bjep.70001

**Published:** 2025-06-10

**Authors:** Richard Göllner, Rebecca Lazarides, Philipp Stark

**Affiliations:** ^1^ Department of Education University of Potsdam Potsdam Germany; ^2^ Cluster of Excellence Science of Intelligence Technical University Berlin Berlin Germany; ^3^ Hector Research Institute of Education Sciences and Psychology University of Tübingen Tübingen Germany

**Keywords:** GPT‐4, lesson transcripts, mathematics lessons, teaching quality

## Abstract

**Background:**

Existing conceptions of teaching quality assume that classroom interactions serve as the foundation for effective teaching. The resulting data necessitates analytical approaches capable of extracting the semantics of these interactions.

**Aim:**

This study investigates whether and to what extent lesson semantics provide insights into teaching quality (i.e., cognitive engagement, encouragement and warmth, multiple approaches, and the nature of discourse). To achieve this, GPT‐4 was applied as a tool for analysing lesson transcripts.

**Sample:**

The study is based on data from the TALIS Video study, which included *N* = 50 teachers delivering two consecutive mathematics lessons in 9th grade. Teaching quality was annotated by trained observers across multiple dimensions.

**Method:**

The analysis involved embedding segmented lesson transcripts to examine their semantic characteristics and associations with human annotations of teaching quality. Additionally, we applied content‐informed prompting to evaluate the interpretability of semantic characteristics for the considered dimensions.

**Results:**

GPT‐4 identified five distinct semantic representations of transcripts, varying at both the teacher and lesson levels. These representations were related to teaching quality, accounting for up to 20% of variance in teaching quality annotations. Content‐informed prompting aligned lesson segments more closely with semantic representations, supporting their interpretability.

**Conclusion:**

The findings suggest that lesson semantics serve as indicators of teaching quality, offering a promising approach to understanding effective classroom learning.

Teaching quality is a crucial factor in explaining students’ motivation and learning achievements. This holds true even when considering students’ learning backgrounds and other relevant characteristics related to the school environment and individual students (Bell et al., 2019; Kane & Staiger, 2012; Klette & Blikstad‐Balas, 2018).

Scholars agree that the essence of teaching quality revolves around classroom interactions between teachers and students, which should be directly related to the learning content, foster a supportive learning environment, and encourage cognitive engagement (CE) (e.g., Pianta et al., 2012). How teachers structure and shape discourse, provide feedback, and frame instructional content is assumed to directly influence students’ comprehension and engagement during classroom learning (Alqassab & León, 2024; Falcon et al., 2023; Putwain et al., 2023). However, these complex interactions result in challenging data that demand new analytical methods beyond those currently used in teaching quality research. Newly available technologies offer promising opportunities for analysing lesson semantics and their relevance to key dimensions of teaching quality.

In this study, we used GPT‐4 to investigate whether and to what extent lesson semantics represent teaching quality in terms of well‐known quality dimensions. Drawing upon the German TALIS Video study (Grünkorn et al., 2020), we examined whether semantic differences exist between teachers and their lessons and explored the extent to which lesson semantics correlate with human annotations of teaching quality. As with other exploratory approaches, interpreting the results is a crucial step. Consequently, we further applied a procedure to more directly assess whether the identified semantic representations of lessons reflect teaching quality.

## TEACHING QUALITY

Teaching quality is commonly understood as the professional behaviour of teachers and their ability to facilitate meaningful learning interactions within a classroom (Doyle, 2006; Fauth et al., 2020; Göllner et al., 2020; Hamre & Pianta, 2010; Kunter et al., 2013).

These interactions are characterized by key qualities including a structured and non‐disturbing lessons course (classroom management), respectful, sensitive, and motivating teaching (learning support), and the use of cognitively challenging tasks (cognitive activation, also labelled as instructional support). Our question is, how these dimensions and the related social interactions manifest in lesson semantics, which refers to how meaning is constructed through language in classroom interactions (e.g., Goodwin et al., 2021; Schleppegrell, 2004). This encompasses not only the explicit content of what is said (such as vocabulary and sentence structures) but also the dynamic processes through which teachers and students collaboratively build understanding. It involves the coherence of discourse, the relational aspects of communication, and the ways in which dialogue shapes learning (Marzano, 2004).

All these elements align with established conceptions of teaching quality (e.g., Pianta & Hamre, 2009), which conceptualize teaching effectiveness as reflected in the nature of classroom interactions. In this vein, it is also reasonable to assume that semantic information is inherently part of any assessment of teaching quality. In addition to other nonverbal cues (e.g., teachers’ body language and the arrangement of pedagogical elements; Babad et al., 2021; Lazarides et al., 2025), classroom observations are likely to implicitly capture aspects of lesson semantics. Due to its verbal and interactional nature, the analysis of lesson semantics may be particularly well‐suited to the dimensions of learning support and cognitive activation, as these are closely linked to teachers’ explanations, discourse facilitation, and emotional responsiveness (Hamre & Pianta, 2010). In contrast, classroom management may rely more heavily on nonverbal behaviours (e.g., gaze direction, movement), which cannot be fully captured through transcript data alone (Martikainen, 2020).

In addition, teaching quality is assumed to manifest at various moments throughout classroom learning (Nolen, 2024). From a professionalization perspective, teaching quality is regarded as a competence that can be acquired and applied consistently across different instructional situations. In contrast, an interactional perspective emphasizes the situational variability of teaching quality, highlighting how it emerges in response to the dynamics between teachers and students. Accordingly, teaching quality is not only expected to vary across lessons (Daumiller et al., 2023) and between classrooms (e.g., Marder et al., 2023; Wagner et al., 2016), but also to fluctuate within a single lesson, shaped by moment‐to‐moment instructional and relational dynamics (Daumiller et al., 2023).

That is, semantic elements are likely fundamental components of teaching quality and warrant further empirical investigation (Hamre & Pianta, 2010; Hou et al., 2024). However, analysing such semantic aspects is a complex task that requires consideration of various interrelated aspects of language and communication during a classroom lesson. Moreover, assessing the semantics of teaching quality cannot be reduced to a single feature; rather, teaching must be viewed as a dynamic system in which multiple elements interact to create an effective learning environment. Given this complexity, we employed a large language model (LLM) to investigate whether lesson semantics could be meaningfully differentiated and to what extent this differentiation aligned with human‐annotated assessments of teaching quality.

## TECHNOLOGY‐ASSISTED ANALYSIS OF TEACHING QUALITY

The use of machine learning, such as LLMs, has become increasingly popular due to their growing accessibility to a wider range of users. These advancements enable more efficient and sophisticated data processing across various research fields. In education, technology‐assisted assessment has been employed to evaluate classroom activities and teaching quality. For instance, a study by Schlotterbeck et al. (2021) used classroom audio data to predict different classroom activities, such as teacher‐led lectures, individual student work, and group work. The audio data captured only paralinguistic aspects of speech (i.e., prosody, number of speakers, and overlapping speech) without converting this information into linguistic content in terms of word meaning. In another study, Gencoglu et al. (2023) applied machine learning to analyse large‐scale open‐ended student responses. Using a topic modelling approach, the authors identified distinct clusters of teaching behaviours based on students' evaluations of teaching quality and developed methods to categorize these responses. The findings demonstrated that technology could enhance assessment processes, achieving performance levels comparable to those of traditionally used assessment methods and procedures (see also Falcon et al., 2023).

The present study investigated lesson semantics related to teaching quality using OpenAI's GPT‐4 model and its text embedding. Technically, GPT‐4 is built using unsupervised learning on language data from extensive sources, processed within an artificial neural network (Brown et al., 2020; Radford et al., 2019). Numerous studies highlight GPT‐4's capability to generate cohesive responses and engage in human‐like conversations (Floridi & Chiriatti, 2020). Additionally, GPT‐4 can analyse texts based on both linguistic and conversational features (see Demszky et al., 2023; Kasneci et al., 2023 for an overview), going beyond simple word occurrences and superficial text features to offer deeper insights into text semantics (Buduma et al., 2022). Most importantly, for the purposes of this study, these methodologies enable the extraction of data that can be further analysed using well‐established social science research methods. This allows us to integrate these methodologies as new powerful attempts to understand teaching quality from a text‐driven perspective (Devlin et al., 2018).

However, as with other exploratory analyses, interpreting the results of machine learning models requires a process of meaning‐making (e.g., Behrens & Yu, 2003). That is, demonstrating that a model can accurately predict an outcome is not sufficient; it is also necessary to understand whether the model relies on information that is theoretically expected to be relevant. As with traditional social science methods, findings derived from machine learning must align with theoretical frameworks to ensure their validity. In the context of language models, meaning‐making is particularly challenging, as one must determine the appropriate level of language information on which to ground interpretation. Considering model outputs at the lowest level (e.g., letters, words, or simpler linguistic patterns) is often too fine‐grained to establish meaningful theoretical connections. Conversely, focusing on higher level features may lead to overly broad interpretations that obscure the most relevant aspects.

In the present study, we approached the meaning‐making process in two ways. First, in analysing the semantics of teaching quality, we required that the results not only distinguish between different teachers but also be sensitive enough to detect variation within and across individual lessons. Second, we applied an approach that allows for novel ways of assessing the meaningfulness of results. Specifically, we used LLMs to generate lesson transcripts that emphasize particular dimensions of teaching quality. These generated texts serve as reference points for evaluating whether the machine learning models capture semantically meaningful aspects of the specific teaching quality dimension under investigation (Cao et al., 2023; Hu et al., 2023; Shin et al., 2020).

## THE PRESENT STUDY

In the present study, we analyse lesson semantics by applying OpenAI's zero‐shot GPT‐4 model to identify similarities among lesson transcripts. Specifically, we will test whether semantic representations derived from the model's text embeddings differ both between lesson segments, lessons as well as teachers. In addition, we will examine the relationship between semantic representations and human‐annotated teaching quality. Finally, we will explore the extent to which semantic information provided by GPT‐4 reflects the specific teaching quality dimensions. The following four hypotheses have been proposed:

First, we expected GPT‐4 and its underlying LLM to provide semantic representations of segmented transcripts which can then be used for further analysis (Hypothesis 1). We apply its text embeddings to convert text input into a numerical format, which is then used to exert components to represent the semantic information of lessons transcripts.

Following the situative turn in educational psychology (Nolen, 2024), we further expect that semantic information extracted from lessons will vary across situations (i.e., 16‐min segments), lessons, and teachers (Hypothesis 2a). Additionally, we hypothesize systematic relationships between extracted semantic information and human‐annotated teaching quality (Hypothesis 2b).

Finally, we examined the extent to which lesson semantics directly reflect teaching quality dimensions. Specifically, we applied a prompting procedure to synthesize transcripts based on different teaching quality dimensions. We expected that the content‐prompted lesson transcripts would align more closely with the extracted semantic representations (Hypothesis 3), thereby providing insight into the validity of these representations in capturing aspects of teaching quality.

## METHODS

### Sample

The present study utilized data from the German TALIS Video Study (Grünkorn et al., 2020) which was funded by the Leibniz Association (2017–2020) in the context of the international TALIS study (Teaching and Learning International Survey; OECD, 2020). The study's primary objective is to investigate effective teaching practices in math for 8th and 9th‐grade students regarding quadratic equations. For this, instructional materials, classroom recordings, as well as student and teacher reports were used to assess teaching quality and its relationship with students’ learning in mathematics. The German sample, which consists of *N* = 50 teachers from 38 secondary schools and 1,140 students from seven federal states, was used for the current study. The participating schools were from academic‐track (Gymnasiums) or vocational‐track schools (Realschule). All participating teachers were asked to conduct two successive lessons to teach quadratic equations to the same class, resulting in two recorded lessons for each teacher and classroom.

### Measures

To address the research questions, we will make use of two different sets of information: linguistic and conversational information from the lesson transcripts and observational ratings of teaching quality based on human annotations of the videotaped lessons. Whereas the observational ratings stem from the national TALIS dataset, the linguistic and conversational information reflect the building block on which the analysis with GPT‐4 had been conducted. Below, we present the set of measures we used in detail:

#### Transcripts

Every spoken word during the lesson was systematically transcribed by watching the video recordings of the lessons. The transcription was already provided by the Research Data Centre Education (FDZ Bildung). Transcriptions for every lesson have been created to support the human annotations of videotaped lessons. The provided transcripts contained anonymous information about the speaker (S#: student(s) number #, T: teacher), the starting time of the utterance, and the utterance itself. One utterance consisted of one or more consecutive sentences spoken by the same speaker (T or S#).

For the analysis, the transcripts were divided into 16‐minute segments corresponding to the rating segments used for annotating teaching quality. We ensured that utterances from the same speaker remained completely intact. However, since we only had access to the start times of individual utterances in the transcripts, long utterances by a single speaker could occasionally exceed the 16‐minute limit by a few seconds. Each 16‐minute segment was then further split into parts consisting of ~500 tokens, evaluated using the tiktoken library (https://github.com/openai/tiktoken). Every text was only ever split at the end of a sentence or at the end of a speaker's statement to preserve linguistic coherence. This additional segmentation was performed for two reasons: first, due to the token limitations of the GPT API; and second, because the specificity of embeddings for longer texts remains uncertain (Cao, 2024) .

#### Measures of teaching quality

In line with existing conceptions of teaching quality, the TALIS video study (Grünkorn et al., 2020) operationalized teaching quality along multiple quality dimensions. In the present study, we focused on dimensions of learning support and cognitive activation. Specifically, for the domain of cognitive activation, we addressed multiple approaches (i.e., focusing on the various approaches students use to solve problems, rather than multiple solutions they generate) and students’ cognitive engagement (i.e., teachers provide students with opportunities to engage in subject‐matter practices). For the domain of learning support, we focused on the nature of discourse (i.e., students are given opportunities to actively participate in classroom discussions) and encouragement and warmth (i.e., the teacher and/or peers provide encouragement to students throughout their learning processes). These quality dimensions were expected to be related to linguistic and conversational information provided by lesson transcripts. According to the rating protocol, two raters independently annotated these teaching quality dimensions. Raters provided annotations for each of the 16‐minute intervals using four‐point scales per indicator, resulting in ratings of three segments per lesson. The reliability of the averaged ratings per segment (ICC[2]) was .63, .65, .70, and .69, respectively, suggesting that measures of teaching quality at the segment level showed at least acceptable precision.

### Analysis

#### Text embedding

All text‐related analyses were performed using Python 3.13. The 500‐token lesson segments were processed using OpenAI's text‐embedding model ‘text‐embedding‐3‐large’ (https://openai.com/index/new‐embedding‐models‐and‐api‐updates/) via the API. An embedding model transforms text into a numerical vector, encoding meaningful information from a text as numerical values within the vector. OpenAI's embedding models have been shown to effectively capture contextual and semantic information across diverse applications, including large‐scale text retrieval and representation tasks (Neelakantan et al., 2022). Studies have demonstrated their ability to perform robust semantic similarity assessments and document separation tasks, aligning with our approach to analysing teaching quality through lesson transcripts (Reimers & Gurevych, 2019). The text‐embedding‐3‐large model generated a 3072‐dimensional vector for each text part, capturing contextual and semantic information from the given text.

After computing the embedding vectors for all 500‐token segmented lesson transcripts, we used Scikit‐learn's StandardScaler function, which normalizes features by subtracting the mean and scaling to unit variance. To reduce dimensionality, we applied principal component analysis (PCA; Andrews, 2016; Buitinck et al., 2013) to the text‐segment embedding vectors. As an unsupervised dimensionality‐reduction technique, PCA projects the high‐dimensional space onto a set of orthogonal, lower dimensional components. In the context of text embedding, this method has been shown to condense the data into compact representations while still preserving important semantic information (Zhang et al., 2024). The number of principal components was determined using the elbow method based on the error sum of squares (Nainggolan et al., 2019). The resulting component scores were calculated for each segment and subsequently used as predictors in the multilevel regression models.

#### Establishing the relationship between semantic representations and teaching quality

To further investigate the association between semantics and teaching quality, we applied a multilevel analytical approach using SAS software (SAS Institute Inc, 2024). A two‐level model was specified, with differences between teachers modelled at the higher level and differences between transcript segments at the lower level. As only two lessons were recorded per teacher, we accounted for the nesting of lessons within teachers by including a dummy variable to control for lesson‐level effects (Snijders & Bosker, 2011). The multilevel prediction model was implemented in two steps. First, we estimated variance components attributable to the different levels using a null model. Second, we included the semantic representations as predictors in a regression analysis to estimate their association with human‐annotated teaching quality. Semantic representations at the lesson and teacher levels were obtained by aggregating the corresponding segment‐level scores in their manifest form. All variables were group‐mean centred at their respective analytical level. This approach allowed us to assess the extent to which the semantic representations explained variation in teaching quality across segments, lessons, and teachers. Due to the number of variables, we tested the associations separately for each of the teaching quality dimensions. Given the relatively small number of teachers and lessons, we also opted not to include random slope parameters. This decision was further supported by our assumption that the relationships between semantics and human ratings would not systematically vary across lessons or segments. Regression results are reported in standardized form, and explained variance in teaching quality was calculated relative to the total variance in each respective teaching quality dimension.

#### Prompting teaching quality

To support the meaning‐making process of the extracted semantic representation, we generated synthetic lesson segments, modifying them to align with the investigated teaching quality dimensions. Specifically, we make use of GPT‐4's generative capabilities, which, however, were applied independently of the text embedding analysis used for PCA. We instructed GPT‐4 to adjust transcript segments based on definitions derived from the TALIS study report, the same definitions used by human coders when assessing teaching quality in fully videotaped lessons (OECD, 2020). For each of the four teaching quality dimensions, we formulated specific prompts to guide the adaptation of the original transcripts in the intended direction. The model was instructed to analyse each lesson transcript sentence by sentence and rewrite the segment according to the respective teaching quality prompt from the codebook. The prompts used for each teaching quality dimension are presented in Table 1. For automated processing, we employed the GPT API (https://platform.openai.com/docs/models), using GPT‐4 with the temperature parameter set to zero to ensure consistency. GPT‐4 has been used and the temperature parameter has been set to zero.^1^ We applied this procedure to 92 randomly selected segments, representing 25% of the available data. Finally, we examined whether the content‐prompting of a specific teaching quality dimension resulted in more pronounced differences of the adjusted segments compared with non‐selected segments across the revealed PCA components. To prevent circularity between the PCA and the generative approach, the adjusted texts were not included in the PCA before.

**TABLE 1:** Text prompts for adjusting lesson transcripts according to the respective teaching quality dimension.

Dimension	Prompt
Multiple approaches (MA)	‘Gehe das folgende Unterrichtstranskript Satz für Satz durch. Schreibe das Unterrichtsgespräch so um, dass die Lehrkraft (L) unterschiedliche Lösungswege vorstellt, Aufgaben auf unterschiedliche Weise bearbeitet werden und Lösungsansätze miteinander verglichen werden. Hier ist das Transkript: …’ ‘Go through the following lesson transcript sentence by sentence. Rewrite the classroom dialogue so that the teacher (T) presents different solution methods, approaches tasks in various ways, and compares different problem‐solving strategies. Here is the transcript: …’
Nature of discourse (ND)	‘Gehe das folgende Unterrichtstranskript Satz für Satz durch. Schreibe das Unterrichtsgespräch so um, dass die Lehrkraft (L) die Schüler (S) am Unterricht beteiligt, eine Interaktion zwischen der Lehrkaft und den Schülern stattfindet und gemeinsam diskutiert wird. Hier ist das Transkript: …’ ‘Go through the following lesson transcript sentence by sentence. Rewrite the classroom dialogue so that the teacher (T) engages the students (S), fosters interaction between the teacher and students, and facilitates a collaborative discussion. Here is the transcript: …’
Encouragement and warmth (EW)	‘Gehe das folgende Unterrichtstranskript Satz für Satz durch. Schreibe das Unterrichtsgespräch so um, dass die Lehrkraft (L) lobt, höflich und geduldig ist und insgesamt ein respektvoller Um‐ gang herrscht (auch unter den Schülern (S)). Hier ist das Transkript: …’ ‘Go through the following lesson transcript sentence by sentence. Rewrite the classroom dialogue to ensure that the teacher (T) offers praise, is polite and patient, and fosters a respectful environment overall (including among the students (S)). Here is the transcript: …’
Students' cognitive engagement (CE)	‘Gehe das folgende Unterrichtstranskript Satz für Satz durch. Schreibe das Unterrichtsgespräch so um, dass die Lehrkraft (L) zum Nachdenken anregt und die Schüler aktiviert. Die Schüler (S) sollen Fragen stellen, kritisch sein und sich für den Stoff begeistern. Hier ist das Transkript: …’ ‘Go through the following lesson transcript sentence by sentence. Rewrite the classroom dialogue so that the teacher (T) encourages critical thinking and actively engages the students. The students (S) should be asking questions, thinking critically, and showing enthusiasm for the material. Here is the transcript: …’

#### Evaluating the prompted texts

In the final step, we applied the embedding model to the prompted lesson segments, used the same scaling, and applied the previously implemented PCA model to the new embedding vectors of the adjusted segments. This resulted in new component scores for each selected content‐prompted segment. To test whether prompting led to changes in the scores, we conducted both univariate (ANOVA) and multivariate (MANOVA) comparisons, accounting for the nesting of segments within lessons and teachers. In general, univariate tests assessed each individual semantic component, while multivariate tests examined the semantic components in conjunction. Two different sets of comparisons were applied: First, we compared selected and non‐selected segments before prompting to determine whether the random selection process was successful. Second, we compared the original and synthesized segments to assess whether differences in component scores were attributable to the prompting procedure. As the selection of texts was applied at the segment level to control for potential teacher differences, the comparisons were conducted solely at the segment level and could not be performed at the lesson or teacher level.

All statistical tests were conducted two‐tailed and with an α‐level of .05.

## RESULTS

### Semantic representation of lesson transcripts

**FIGURE 1:**
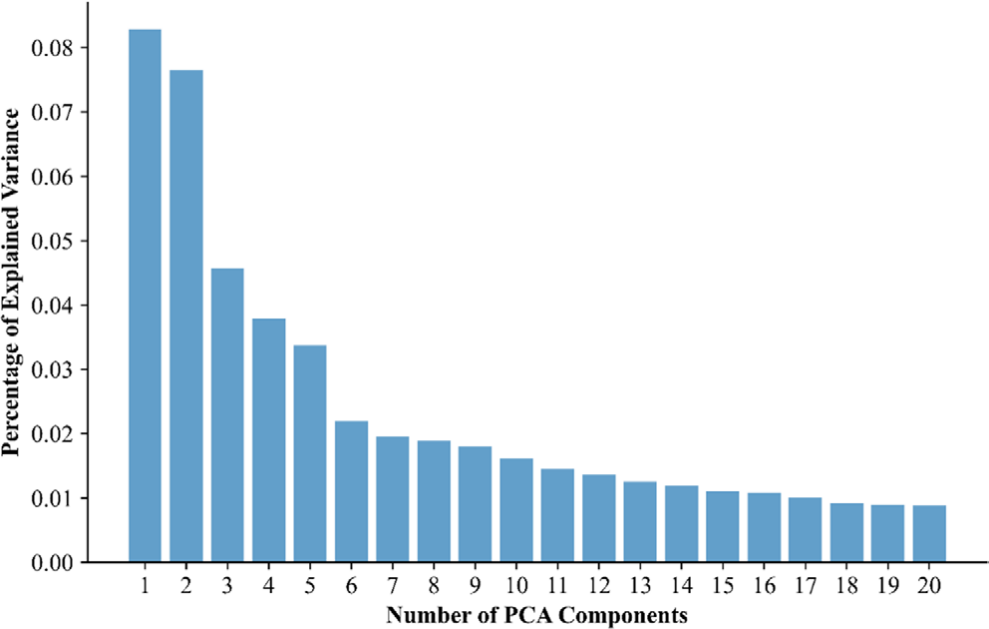
Elbow plot showing the percentage of explained variance in GPT‐4 embeddings for the first 20 components.

Addressing the first hypothesis, we analysed the embeddings of transcripts to examine whether they can be characterized by their semantic properties. PCA revealed a solution consisting of five orthogonal components (see elbow plot, Figure 1), suggesting that five components adequately capture the semantic information of transcript segments comprising 500 tokens.

### Associations of semantic representations with human annotations of teaching quality

Regarding the hypothesis that semantic representations of lesson segments were associated with human annotations of teaching quality (Hypothesis 2a), multilevel regression models were applied. We began with a model that included no predictors to determine the extent to which semantic representations varied between segments, between lessons, and between teachers. The results revealed the following findings: First, component scores of semantic representations revealed consistency within lessons as well as some consistency within teachers. On average, 2% to 13% of the variance (median = 12%) in semantic information could be attributed to differences between teachers and 3% to 52% of the variances (median = 37%) could be attributed to lessons showing a semantic consistency within lessons and across the analysed transcript segments. The corresponding results for human annotations showed that 9% to 32% of the variance (median = 21%) in human annotations could be attributed to teacher differences and 11%–33% of the variance (median = 23%) in human annotations could be attributed to the consistency of ratings within lessons.

We then used PCA scores to predict human‐annotated teaching quality at the segment, lesson, and teacher level (Hypothesis 2b). Table 2 presents the results for each of the quality dimensions. The results showed that semantic representations were associated with teaching quality at all levels, whereby the associations were particularly close for cognitive engagement (.08 ≤ *R* ^2^ ≤ .20) and nature of discourse (.14 ≤ *R* ^2^ ≤ .16). In contrast, the predictive power was lower for multiple approaches (.01 ≤ *R* ^2^ ≤ .06) as well as encouragement and warmth (.02 ≤ *R* ^2^ ≤ .17). Inspecting the single components, the results showed that teaching quality dimensions were each predicted by separate components at the different analytical levels. Additionally important, except for multiple approaches, the semantic components explained variations of teaching quality at the teachers’ level. That is, semantic information derived from lesson transcripts provided information to differentiate between teachers in terms of their annotated teaching quality.

**TABLE 2:** Multilevel models predicting human annotations of teaching quality with semantic components.

	Cognitive engagement			Encouragement and warmth			Multiple approaches			Nature of discourse		
	*β*_S_	*β*_L_	*β*_T_	*β*_S_	*β*_L_	*β*_T_	*β*_S_	*β*_L_	*β*_T_	*β*_S_	*β*_L_	*β*_T_
SR1	−.24***	−.14**	−.34***	.03	−.01	−.18*	−.01	.00	−.05	−.16***	−.09*	−.25*
SR2	.03	.06	.08	.03	.01	−.32***	.04	.06	.11	−.19***	−.19***	−.14
SR3	.20***	.17***	.18**	.10*	.08*	.07	.00	.00	.19*	.26***	.24***	.15
SR4	.11**	.06	−.04	.14***	.09*	−.03	−.11*	−.07	−.13	.11*	.07	−.15
SR5	.15***	.12**	.13*	−.02	−.06	−.18*	0.04	.03	−.07	−.16***	−.14***	−.19*
Lesson		.14			.03			−.08			.20*	
*R* ^2^	.17	.08	.20	.03	.02	.17	.02	.01	.06	.16	.14	.15

*Note*: Lessons were dummy coded, with the first lesson serving as the reference category. Due to the number of independent variables and the resulting model complexity, associations were tested separately for each teaching quality dimension. For presentation purposes only, the associations between the semantic representations and the teaching quality annotations at the different levels are shown side by side. *R* ^2^ was computed as a pseudo‐*R* ^2^, referring to the proportion of total variance explained in the respective dependent variable.

Abbreviations: L, lesson‐level model; S, segment‐level model; SR, semantic representations; T, teacher‐level model.

*** *p* < .001;

** *p* < .01;

* *p* < .05.

### Explaining semantic representations using prompted transcript

**FIGURE 2:**
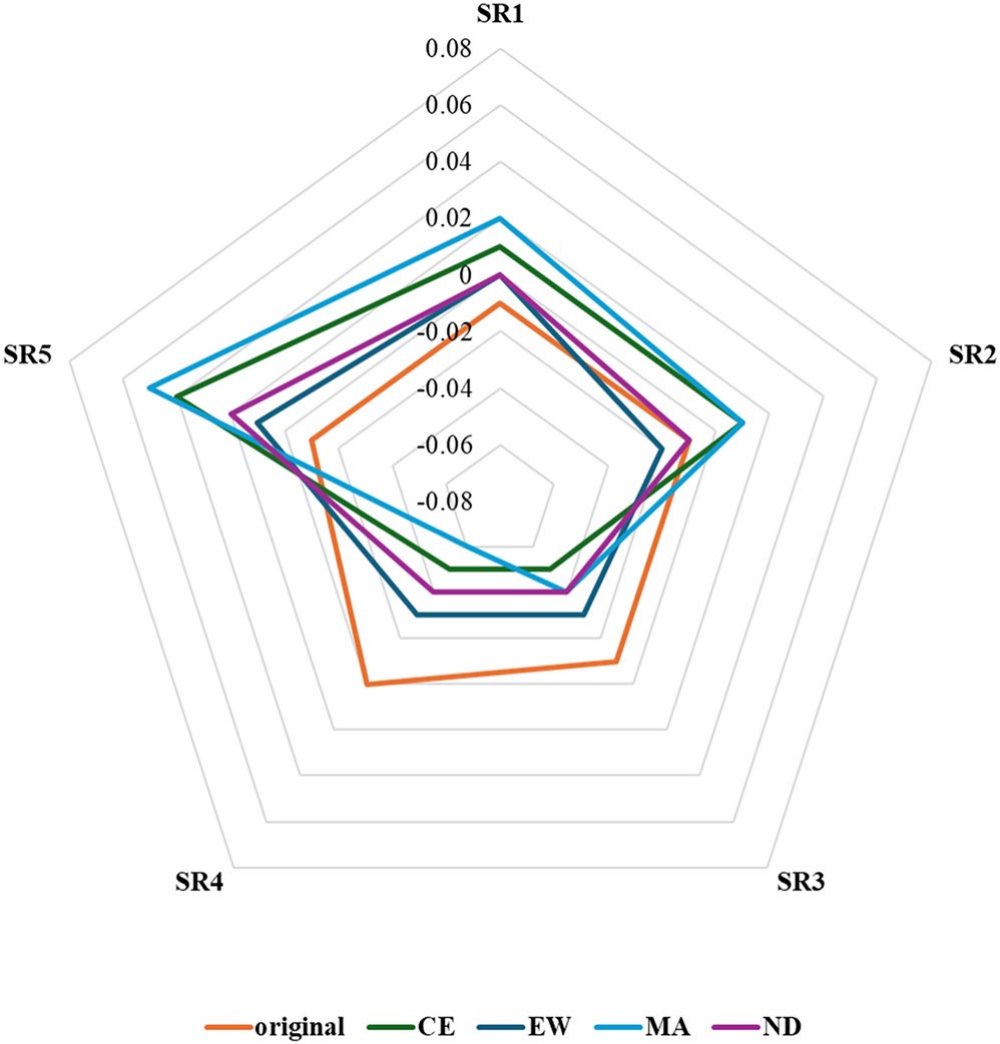
Depiction of configurations of semantic representations before and after the prompting of cognitive engagement (CE), encouragement and warmth (EW), multiple approaches (MA), and nature of discourse (ND).

Turning to our last hypothesis (Hypothesis 3), 25% of randomly selected segments were prompted to examine whether and to what extent semantic representations represent the semantic information of lessons in relation to the teaching quality dimensions in question. To achieve this, we compared the revealed representations before and after prompting the transcript segments. The resulting lesson segments demonstrated face validity (see https://osf.io/z2tcq/?view_only=d2e49a98248e46a98e2a8837f391100c) and the number of tokens did not statistically significantly differ (*p* = .21) between the original (*M* = 587.82, *SD* = 141.59) and synthetic segments (*M* = 577.57, *SD* = 137.98). Furthermore, means of components did not reveal a difference between selected and non‐selected segments before prompting (original; Table 3). Neither univariate comparisons of the semantic components (*p*s > .05) nor a multivariate comparison of all components together showed a statistically significant difference, *F*(5, 373) = 1.53, *p* = .179, *η* ^2^ = .02. In line with the hypothesis, the comparison of synthetic segments (Table 3) showed more accentuated mean differences in comparison to the original segments. All multivariate tests revealed statistically significant differences between original and synthetic transcripts, indicating that semantic information represented by the revealed semantic representations can be influenced through prompting based on a theoretically defined quality dimension. Moreover, the results of univariate comparisons showed that prompting did not lead to differences across all semantic representations but was limited to specific ones. Specifically, we found less pronounced differences for the first and second components, along with some homogeneity of effects for the remaining three components (see also Figure 2). This suggests that the prompting procedure did not influence all semantic representations equally. Instead, the semantic shaping of lesson segments was more nuanced and less uniformly distributed across components than initially expected.

**TABLE 3:** Summary of mean differences between original and synthetic transcript segments.

	Original		Synthetic			
	Selected	Non‐selected	Cognitive engagement	Encouragement and warmth	Multiple approaches	Nature of discourse
SR1	−.01	.01	.01	.00	.02	.00
SR2	−.01	.01	.01	−.02*	.01	−.01
SR3	−.01	−.01	−.05***	−.03**	−.04***	−.04***
SR4	.00	.00	−.05***	−.03**	−.06***	−.04***
SR5	−.01	.01	.04***	.01	.05***	.02
Wilk's Lambda		*F*(5, 373) = 1.53, *p* = .179, *η* ^2^ = .02	*F*(5, 373) = 14.32, *p* < .001, *η* ^2^ = .16	*F*(5, 373) = 4.96, *p* < .001, *η* ^2^ = .06	*F*(5, 373) = 17.72, *p* < .001, *η* ^2^ = .19	*F*(5, 373) = 7.63, *p* < .001, *η* ^2^ = .09

*Note*: Results presented factor means. All comparisons refer to the means of the selected transcript segments before prompting. Univariate tests were complemented with a multivariate test (Wilk's Lambda) to compare the configuration of semantic representations before and after prompting. ***p < .001; **p < .01; *p < .05.

## DISCUSSION

The present study examined whether and to what extent the semantics of lesson transcripts in math lessons reflect multiple dimensions of teaching quality. Using GPT‐4, transcripts were analysed based on a range of semantic information, from syntactical to pragmatic and conversational features, that shape teaching and learning interactions. The study findings demonstrated that the semantic representations of lessons align with teaching quality.

First, the revealed semantic representations were shown to be sensitive enough to reflect variations across segments, lessons, and teachers, indicating that lesson semantics sensitively capture differences in ongoing teaching. These findings align closely with existing conceptions of teaching quality. In contemporary frameworks (e.g., Daumiller et al., 2023; Nolen, 2024), teaching quality and learning responses are no longer viewed as a static construct defined solely by a teacher's ability to provide effective instruction. Instead, it is understood as a dynamic process that manifests in teacher–student interactions and evolves throughout a lesson, which consists of various learning situations. The finding that semantic information varies across teachers, lessons, and segments highlights the potential of modern technologies to capture this complexity. It demonstrates how advanced analytical approaches can assist the assessment of teaching quality on the basis of semantic information (Demszky et al., 2023; Hou et al., 2024).

Second, the present findings showed that the revealed semantic representations of lesson transcripts were associated with human‐annotated teaching quality dimensions. Prediction models indicated a significant amount of explained variance at different levels. Specifically, semantics accounted for up to 20% of the variation in human annotations across different quality dimensions. Most importantly, variance was explained at all levels, with the strongest effects observed at the teacher level. This finding supports the notion that semantic representations can effectively detect differences in teaching quality between teachers (Goodwin et al., 2021). When interpreting these findings, it is important to note that human raters were not instructed to focus solely on the linguistic or semantic aspects of the lessons. Instead, they were asked to assess teaching quality based on all available information in the videotaped lessons, including nonverbal behaviour and the arrangement of pedagogical elements. Within this context, the observed associations underscore the relevance of lesson semantics as a relevant component of effective classroom learning. The study's focused examination of semantic information, therefore, enables a more nuanced investigation of an important yet underexplored aspect of teaching quality from both scientific and practical perspectives (e.g., Leiss et al., 2019). Notably, the strongest associations between lesson semantics and teaching quality were found for students’ cognitve engagement and the nature of discourse. While these two quality dimensions are understood as aspects of classroom interaction within the TALIS framework, the dimension of multiple approaches may be more closely related to task design and the use of learning materials. In the case of encouragement and warmth, one could argue that paraverbal and nonverbal information which was not captured in the analysis of lesson transcripts may also play a significant role (Lazarides et al., 2025). In this vein, the study findings demonstrate that GPT‐4 can effectively analyse conversational data and extract key indicators of teaching quality.

Finally, technology‐assisted research, as conducted in the present study, still faces significant challenges. A particularly important issue is the need for additional steps to interpret empirical findings within a theoretical framework. To address this, we applied a structured procedure to enhance our understanding of the revealed associations between semantic information derived from lesson transcripts and teaching quality. Specifically, we used GPT‐4's capabilities to generate synthesized data, allowing us to explore the significance of lesson semantic representations. By creating synthetic transcripts that reflected theoretically derived variations in lesson quality dimensions, we examined whether the derived semantic representations of lessons accurately captured specific teaching quality attributes. Our study demonstrated that GPT‐4‐generated transcripts were face‐valid representations of typical secondary school mathematics lessons and that synthesized transcripts did not systematically differ from the originals in terms of average text length. Furthermore, content prompts effectively modified the lesson transcripts in alignment with the revealed semantic representations. Component scores became more pronounced for specific components, suggesting that the extracted semantic information reflects aspects of the targeted teaching quality dimension. In this regard, the findings indicate that the identified semantic components contained relevant information about teaching quality, rather than being merely influenced by construct‐irrelevant features of the analysed transcripts.

On the other hand, our findings suggest that the transcript adaptations were less distinctive than initially expected. While the multivariate analyses indicated that segments differed collectively across all semantic components, the univariate results showed that the adaptations produced similar shifts, limited to only some of the extracted components. Ideally, the prompting was expected to elicit more specific combinations of components that would reflect the uniqueness of each targeted teaching quality dimension. However, this was not the case, raising the question of what specific types of semantic information were actually represented by the extracted semantic components. Answering this question requires further research that might apply a similar methodological approach as used in the present study, but vary the granularity of the prompts. By systematically manipulating the specificity and scope of the prompt instructions, future studies may be able to determine which types of semantic content related to teaching quality are being encoded in lesson transcripts.

### Limitations and further research

In general, the present study provides insights into a semantic analysis of lesson transcripts and their associations with teaching quality dimensions. The text embedding and clustering of transcripts revealed semantic representations that varied both within and between lessons and were associated with human annotations of multiple teaching quality dimensions. While the approach successfully identified semantic representations related to human annotations of teaching quality, we acknowledge the potential limitations of this study. These limitations are closely tied to the use of machine learning in extracting meaningful characteristics that inform specific semantic features of effective classroom learning. The inherent ‘black‐box’ nature of these models presents a challenge, underscoring the need for robust theoretical frameworks to guide interpretation and meaning‐making. Furthermore, OpenAI's GPT models are not open source, which prevents us from following the processing procedures in the model. Additionally, it is essential to consider that we employed a zero‐shot GPT model rather than a model specifically trained on human‐labelled teaching quality data. The latter could potentially yield higher accuracy in predicting teaching quality. Furthermore, developing new models could incorporate relevant additional features. One particularly important aspect is the variability in human ratings of lessons, which often exhibit a degree of disagreement (White & Ronfeldt, 2024). Extending models to account for such discrepancies in human annotation could improve predictive accuracy by integrating measures of uncertainty and variability, thereby enhancing the robustness of current scaling procedures used in the field.

Furthermore, one might assume that semantic representations may overlap with pedagogical elements (e.g., varying classroom activities) that are typically considered aspects of classroom organization rather than indicators of teaching quality. In this vein, the extent to which the revealed semantic representations are related to specific pedagogical elements remains an open question. Addressing this issue in future research may offer valuable insights into the interactional patterns that emerge through the orchestration of pedagogical elements, thereby contributing to a more nuanced understanding of how teaching quality is enacted in real classroom settings.

Another challenge concerns the approach used to critically assess the meaning of semantic representations. While we believe that using synthetic data based on theoretical definitions is a promising strategy for deriving insights from model results, the present study did not aim to systematically investigate this procedure. Indeed, the findings cannot confirm that the synthetic data ultimately reflect the specific teaching quality dimension in question. Using human‐generated transcripts would provide greater assurance that the content offers a valid representation of teaching quality. Additionally, one might consider a study comparing human ratings of teaching quality across original and synthetic transcripts to explore systematic differences introduced through the synthetic generation process. Even when accepting the procedure of the present study, its practical implementation involves several considerations. In particular, the formulation of prompts guiding the model to generate lesson transcripts according to a specific definition is critical. Even for well‐established definitions of teaching quality, numerous model parameters must be carefully specified, including the required length of synthetic transcripts, the level of detail regarding classroom behaviours or events, the degree to which the adapted transcript may diverge from the original, and the optimal amount of text needed to generate sufficiently specific content without compromising the lesson's narrative structure. Finally, it has to be kept in mind that other approaches exist helping to bridge the gap between model outputs and theory‐based interpretation. These approaches range from non‐technically assisted strategies such as expert ratings of model outputs to post‐hoc feature attribution methods helping to identify which features most influenced model predictions. Future research should systematically explore the potentials of the approach used in the present study. Such efforts will be essential to determine whether the proposed strategy can evolve into a more robust approach for validating the results of LLMs in the context of teaching quality research.

## CONCLUSION

The present study investigated the semantics of lesson transcripts in mathematics classrooms and examined whether the extracted semantic information was associated with human‐annotated teaching quality using a zero‐shot LLM. The results demonstrated that lesson semantics are sensitive to differences in teaching quality both within and between lessons as well as across teachers. Additionally, our findings revealed significant associations between lesson semantics and human‐annotated teaching quality, highlighting semantic information as a fundamental and integral component of teaching quality. Finally, the extracted semantic representations were found to reflect construct‐relevant aspects of teaching quality rather than construct‐irrelevant features, thereby underscoring their substantive meaning. At the same time, this raises the question of which specific semantic characteristics actually contribute to fostering teaching quality during regular classroom lessons.

## AUTHOR CONTRIBUTIONS

**Richard Göllner:** Conceptualization; writing – original draft; funding acquisition; methodology; validation; resources; project administration. **Rebecca Lazarides:** Writing – review and editing; investigation; validation. **Philipp Stark:** Formal analysis; data curation; writing – review and editing; visualization; methodology; validation; software.

## CONFLICT OF INTEREST STATEMENT

The authors have no conflict of interest to declare.

## Data Availability

The data that support the findings of this study are available in FDZ at https://www.dipf.de/de/infrastrukturen/forschungsdaten/forschungsdatenzentrum‐bildung. These data were derived from the following resources available in the public domain: FDZ, https://www.dipf.de/de/infrastrukturen/forschungsdaten/forschungsdatenzentrum‐bildung.

## Footnotes

^1^
The temperature parameter influences the probability distribution from which the model selects the next token (word or piece of a word). A higher temperature (e.g., 1.0) makes the output more diverse and random, whereas a lower temperature result in more deterministic and reproduceable output.
